# Merging Electron Deficient Boronic Centers with Electron-Withdrawing Fluorine Substituents Results in Unique Properties of Fluorinated Phenylboronic Compounds

**DOI:** 10.3390/molecules27113427

**Published:** 2022-05-26

**Authors:** Agnieszka Adamczyk-Woźniak, Andrzej Sporzyński

**Affiliations:** 1 Faculty of Chemistry, Warsaw University of Technology, Noakowskiego 3, 00-664 Warsaw, Poland; 2 Faculty of Agriculture and Forestry, University of Warmia and Mazury, Oczapowskiego 2, 10-719 Olsztyn, Poland

**Keywords:** boronic, benzoxaborole, acidity, crystal structure, molecular docking, biological activity

## Abstract

Fluorinated boron species are a very important group of organoboron compounds used first of all as receptors of important bioanalytes, as well as biologically active substances, including Tavaborole as an antifungal drug. The presence of substituents containing fluorine atoms increases the acidity of boronic compounds, which is crucial from the point of view of their interactions with analytes or certain pathogen’s enzymes. The review discusses the electron acceptor properties of fluorinated boronic species using both the acidity constant (p*K*_a_) and acceptor number (AN) in connection with their structural parameters. The NMR spectroscopic data are also presented, with particular emphasis on ^19^F resonance due to the wide range of information that can be obtained from this technique. Equilibria in solutions, such as the dehydration of boronic acid to form boroxines and their esterification or cyclization with the formation of 3-hydroxyl benzoxaboroles, are discussed. The results of the latest research on the biological activity of boronic compounds by experimental in vitro methods and theoretical calculations using docking studies are also discussed.

## 1. Introduction

**Figure 1:**
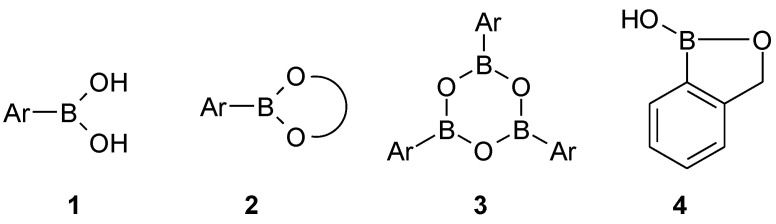
The most important arylboronic species: boronic acids (**1**), cyclic esters (**2**), anhydrides (boroxines) (**3**) and benzoxaboroles (**4**).

Boronic acids and their derivatives (Figure 1) are the object of interest in the dynamically developing field of chemistry. Although they have been known for over 150 years [1,2,3], interest in their unique and useful properties has been growing steadily in the last few decades.

**Figure 2:**
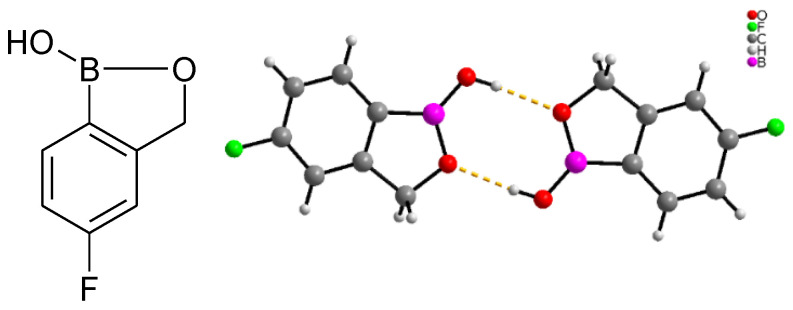
5-Fluoro-1,3-dihydro-2,1-benzoxaborol-1-ol and the crystal structure of its dimer [14].

The reason for this growth is their application in important areas of chemistry. In the history of research involving boronic acids, milestones can be pointed out:

- Already in 1954, Kuivila [4] reported that boronic acids form cyclic esters with cis-diols in a fast and reversible reaction. In 1959, Lorand and Edwards determined the values of the equilibrium constants of ester formations with various sugars [5]. Practical uses of this reaction were developed much later [6], and it has become the basis for the design of molecular receptors of important biomolecules, e.g., glucose, catecholamines or adenosine monophosphate (AMP).
- The Suzuki–Miyaura coupling reaction [7] is the basis for the synthesis of biaryl systems. It was developed as a reaction of boronic acids with aryl bromides catalyzed by palladium compounds and, subsequently, a subject of numerous modifications, extending the range of reagents, solvents and catalysts. The importance of this reaction is evidenced by the fact that searching for the phrase “Suzuki” in the Scopus database in 2020 both in the titles and abstracts gave 1355 responses, as well as a Nobel prize for that reaction acquired in 2010.
- Benzoxaboroles, cyclic hemiesters of phenylboronic acids, have been known since 1957 [8] and received moderate interest until it was found that these compounds bind sugars at a physiological pH [9]. This allowed not only development of a group of receptors of new type but also for the application of these compounds as biologically active substances with antibacterial, antiprotozoal and antifungal activity [10,11,12]. The most famous representative of benzoxaboroles contains a fluorine atom and is used as powerful drug against onychomycosis—Kerydin (Tavaborole) [13] (Figure 2).

Other examples of biologically active fluoro-containing benzoxaboroles are given in [15]. The increased interest in this group of compounds may be demonstrated by comparing the number of benzoxaboroles discussed in two comprehensive reviews. Prepared in 2009, our paper: “Benzoxaboroles—old compounds with new applications” [16] took into account 66 compounds; we did not think that the next review published 6 years later would cover 550 compounds [17].

Currently, boronic compounds are used in all areas of chemistry: organic synthesis as catalysts for the construction of sensors of important analytes and applications in medicine, including antifungal, antibacterial, anticancer and antiprotozoal drugs or probes for detecting reactive oxygen species [18,19]. Their application in materials chemistry is associated primarily with the possibility of creating dynamic systems with coordination bonds, e.g., in self-repairing polymers [20].

The substituents in the aromatic ring of arylboronic compounds significantly affect the properties of these compounds. This effect is based on the change of the electron density in aromatic ring and, as the consequence, on the boron center. Another type of action includes specific interactions of the neighboring functional group with the boronic center. An example may be *ortho*-aminomethylphenylboronic acids, in which the interaction of the nitrogen atom’s lone pair with the boronic group is the basis for the construction of PET-type sugar receptors [21,22,23,24]. Phenylboronic acids with *ortho*-alkoxy substituents are another example where the formation of strong intramolecular hydrogen bonds can lead to the formation of unusual monomeric structures [25]. The influence of *ortho* substituents on the properties of arylboronic acids has been recently reviewed [26].

Boronic compounds containing fluorine substituents constitute a special group with a wide range of applications. The introduction of a fluorine atom directly into the aryl ring or its substituents (e.g., as a CF_3_ or OCF_3_ group) significantly affects the properties of these compounds, primarily by increasing their acidity. Boronic acids generally display lower acidity than their carboxylic analogs. The p*K*_a_ of the unsubstituted phenylboronic acid is 8.86 [27], whereas that of benzoic acid is 4.20 [28]. It is worth noting that the acidic character of most boronic acids is not of a Brønsted acid but usually of a Lewis acid. From the point of view of applications of these compounds, it is advantageous to increase their acidity, which, for example, allows the binding of sugars at a physiological pH or facilitates the binding of anions. Fluorinated organoboron compounds are also unique acid catalysts whose activity is regulated by the number and position of fluorine substituents [29]. Finally, it is worth emphasizing that about 25% of known drugs contain the fluorine atom [30]. Therefore, the combination of a benzoxaborole unit and fluorine substituents in one molecule may result in increased biological activity. Very recently, the history of the use of boron functional groups in medicinal chemistry was reviewed [31]. Two comprehensive reviews on boron-containing heterocycles as pharmacological agents [32] and boron chemicals in drug discovery [33] were also recently published.

Fluorinated arylboronic acids are susceptible to hydrodeboronation. Recently, Lloyd-Jones et al. investigated the kinetics and mechanism of the base-catalyzed hydrolysis and protodeboronation of a series of fluorinated boronic compounds. They found that pinacol boronic esters display a unique stability at a high pH compared to other commonly employed esters [34].

In 2018, a comprehensive review on fluorinated boronic compounds was published [15]. The work included a detailed compilation of the acidity constants of fluoro-substituted boronic acids and benzoxaboroles, the geometric parameters of those molecules based on crystallographic data and NMR data, as well as an overview of the methods of synthesis of the individual groups of compounds and examples of their applications. The issue of the biological activity of fluorine-containing boronic compounds has only been briefly discussed. In recent years, numerous works have been published involving novel boronic compounds with fluorine-containing substituents, e.g., CF_3_ and OCF_3_ groups. Some of them show significant biological activity. The aim of the present review is to compile the results of studies carried out in recent years with a more comprehensive discussion of the biological activity of fluorinated boronic compounds.

## 2. Acidity of Fluorinated Boronic Compounds

**Scheme 1:**
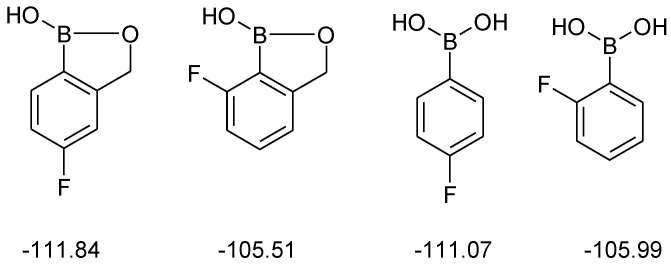
^19^F NMR chemical shifts (ppm) for the selected fluoro-substituted benzoxaboroles and phenylboronic acids.

As already mentioned, the acidity of boronic compounds is a very important parameter characterizing these compounds, and its increase is beneficial from the point of view of numerous applications. The introduction of a fluorine substituent into phenylboronic acids increases their acidity. This effect is, however, dependent on the position of the F substituent. The lowest influence is observed for the *para* position as the result of the compensation of the inductive and resonance effects, which have comparable values [35]. For the *meta* position, the resonance contribution is much weaker, resulting in an increase of the acidity. In the case of the *ortho* derivative, enhanced acidity can be caused by the formation of an intramolecular B–O–H···F hydrogen bond. In most cases, introducing further fluoro substituents causes an increase in acidity, but the position of the substituents also plays an important role. The range of p*K*_a_ values for fluorinated phenylboronic acids is 6.17–8.77, with the lowest value for 2,3,4,6-tetrafluorophenylboronic acid and the highest one for 4-fluorophenylboronic acid [36]. A similar effect is observed for fluoro-substituted benzoxaboroles. An unsubstituted compound has a p*K*_a_ value of 7.39 [37], and the introduction of the fluorine substituent into an aromatic ring reduces this value to 6.36–6.97, depending on the position of the substituent [38,39]. The exception is 7-fluorobenzoxaborole, for which the p*K*_a_ value is 7.42 [38]. The effect is opposite to that of *ortho*-phenylboronic acid, where the acidity is higher than that of the other isomers. The crystal structure of 7-fluorobenzoxaborole has not been studied so far. However, based on the analysis of ^19^F NMR data showing similar chemical shifts in pairs of analogs [38,40], it can be concluded that an intramolecular hydrogen bond is formed in this compound; the chemical shift values are similar to the analogs of boronic acids (Scheme 1).

However, in the case of benzoxaborole, the structure formed would be rigid, which would make anion formation difficult and result in a lower acidity.

Interestingly, fluoro-substituted 1,2-diboronic acids revealed very high acidity (p*K*_a_ 3.0–5.3), which can be explained by the increased stability of the boronate anion by the interactions of two boronic groups [41].

Monofluorophenylboronic acids with the formyl group at the *ortho* position show a significant increase in acidity due to the introduction of a formyl group. This is mainly due to the strong resonance effect of the formyl group in the phenyl ring, decreasing the electron density at the boron center [27].

In recent years, the subjects of our interest were phenylboronic compounds with CF_3_ and OCF_3_ substituents. As already mentioned, in the case of a fluoro substituent, there are two effects: inductive and resonance, displaying opposite directions. For the CF_3_ group, the resonance effect is not observed, and for the OCF_3_ group, it is relatively weak. Those effects are manifested in Hammett constants for various fluorine substituents [42], presented in Table 1.

The above data show that both the CF_3_ and OCF_3_ groups should significantly affect the properties of boronic compounds. Contrary to the F substituent, lowering the electron density of the aromatic ring is also observed for the *para* position. The p*K*_a_ values of the boronic acids possessing these groups are collected in Table 2.

**Scheme 2:**
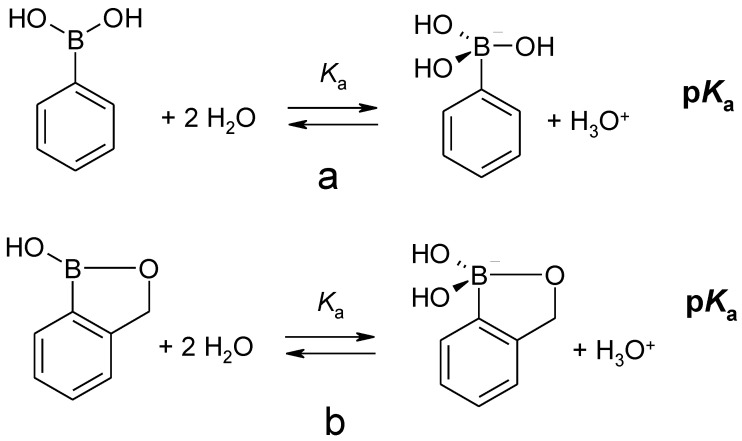

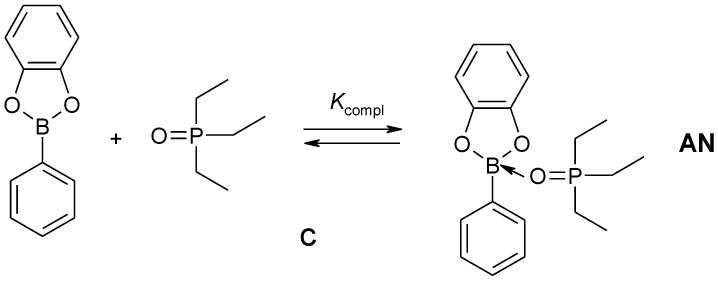
Equilibria for boronic acid (**a**), benzoxaborole (**b**) and boronic ester (**c**) connected with the acidity constant and acceptor number.

The influence of the substituent on the Lewis acidity depends on its position in the phenyl ring. The acidity for the *meta* and *para* isomers with the CF_3_ and OCF_3_ substituents is higher than for the F one, which is consistent with the values of Hammett constants. A significant rise in acidity is observed for CF_3_-substituted acids with an additional 2-CHO substituent. The situation is quite different for compounds with fluorine substituents at the *ortho* position. In the case of the F-substituent, increased acidity is caused by the formation of a B–O–H···F intramolecular hydrogen bond. For the CF_3_ and OCF_3_ groups, a significant reduction in acidity of the *ortho* isomers can be explained by the influence of a bulky substituent proximal to the boronic group due to the steric inhibition of the tetrahedral boronate ion formation (Scheme 2a).

Benzoxaboroles generally show higher acidity compared with the corresponding boronic acids; for unsubstituted compounds, the p*K*_a_ values are 7.39 and 8.86, respectively. Similar differences are observed for mono fluoro-substituted compounds [15]. A comparison of acidity constants for CF_3_-substituted bis-benzoxaborole with other boronic compounds with the CF_3_ group at the *para* position is shown in Table 3.

The Lewis acidity of phenylboronic esters can be described by the value of Gutmann’s acceptor number [48,49,50,51]. The term “acceptor number” (*AN*), initially developed for a quantitative description of the electrophilic properties of solvents, is proportional to the change in ^31^P NMR shift between complexed and uncomplexed triethylphosphine oxide (Et_3_PO). Gutmann defined acceptor number by Formula 1.

(1) $$AN=\frac{\delta_{\mathrm{complex}}-\delta_{1}}{\delta_{2}-\delta_{1}}\cdot 100$$

where $\delta_{\mathrm{complex}}\;$is the ^31^P NMR chemical shift of the Et_3_PO–Lewis acid complex, and $\delta_{1}$ and $\delta_{2}$ are the ^31^P NMR chemical shifts of Et_3_PO dissolved in hexane (41.0 ppm) and SbCl_3_ (86.1 ppm), respectively. The values for hexane (*AN* = 0) and SbCl_3_ (*AN* = 100) are the arbitrarily fixed points within the *AN* scale.

Beckett and coworkers [52] determined the *AN* values for the selected boron compounds. This approach was also used for a quantitative determination of the Lewis acidity of the fluorinated boronic catechol esters. Since the formation of the complex between the ester and Et_3_PO was found to be an equilibrium reaction, Gutmann’s method was modified by the extrapolation of the data to infinite ester excess [53]. Similarly, as in the case of boronic acids, the introduction of fluorine substituents increases the acidity of catechol boronates. In general, the introduction of additional fluorine substituents further increases the acidity.

**Figure 3:**
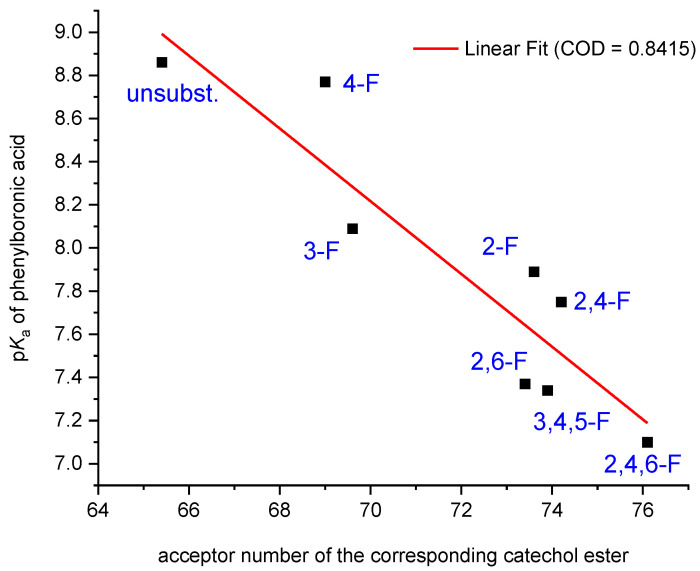
p*K*_a_ of fluoro-substituted phenylboronic acids vs. acceptor number of the corresponding catechol esters.

A linear correlation of the acidity constants of fluorophenylboronic acids with the acceptor number of their catechol esters is shown in Figure 3.

The reactions that are the basis of the acidity constant and the acceptor number determination are shown in Scheme 2.

At it is shown in Figure 3, the relationship between p*K*_a_ and acceptor number does not correlate well even for a series of similar acids and esters This could be due to a much greater steric effect when boronic ester is complexed by the Et_3_PO molecule in comparison with interactions with the hydroxyl ion. It is also worth noting that the structure of a diol part in the ester molecule significantly influences the *AN* value [54].

Recently, the correlation between p*K*_a_ values measured in water for metal–aqua species [M(H_2_O)_m_]^n+^ and the acceptor number in the polar organic solvents was analyzed. The authors obtained a good correlation, which can be explained by the lack of a steric effect in the case of the investigated species [55].

The acceptor number determination is a good tool for the investigation of boron compounds as anion-complexing additives improving the conductance in lithium-ion battery electrolytes. Several boronic esters were investigated in terms of these properties [56,57]. However, the investigated compounds showed a limited impact on the properties of electrolytes. Moreover, it was observed that some esters are not stable in the presence of polar solvents and dissociated ionic species.

## 3. Crystal Structures

### 3.1. Compounds with F-Substituents

The basic structural parameters of fluorinated boronic compounds were collected in the 2018 review [15]. An analysis of these data led to the following conclusions:

- A majority of fluoro-substituted phenylboronic acids form basic dimeric *syn-anti* synthons. The differentiation of the crystal structure occurs at higher-order supramolecular organization.
- In *ortho*-fluorophenylboronic acids, intramolecular B-O-H···F hydrogen bonds are formed. These bonds are weak and practically do not affect the crystal structure. The second *ortho*-fluoro substituent does not form such a bond.
- The molecules are not planar. The twist of the boronic group is observed with the value of the dihedral angle about 25° for most compounds. The introduction of additional substituents into the phenyl ring influence this value considerably.

In the crystal structure of 5-fluoro-1,3-dihydro-2,1-benzoxaborol-1-ol, antifungal drug Kerydin (Figure 2), nearly planar molecules form centrosymmetric dimers via strong O-H···O hydrogen bonds. The dimers are arranged into layers by weak intermolecular C-H···O and C-H···F hydrogen bonds [14]. The crystal structure of the corresponding 6-fluoro isomer is very similar [38].

For the series of fluoro-substituted phenylboronic catechol esters, the influence of the number and positions of fluorine atoms on the molecular and crystal structures was investigated [58]. The substantial differentiation of the dipole moments generated by fluorine substitution and its consequences on molecular packing were analyzed. The presence of *ortho*-fluoro substituents enhances the proton acceptor character of oxygen atoms. Consequently, the C−H···O hydrogen-bonded centrosymmetric dimer is the dominating motif for molecules with small dipole moments. Otherwise, the antiparallel dipole−dipole interactions are responsible for the supramolecular architecture [58].

### 3.2. Compounds with CF_3_ and OCF_3_ Substituents

Crystal structures of three isomers of (trifluoromethyl)phenylboronic acids are described in [43]. All the compounds form typical *syn-anti* hydrogen-bonded dimers. The main difference in the geometrical parameters is observed in a dihedral angle between a plane defined by the phenyl ring and a plane defined by the BO_2_ group. The smallest values of 6.7° and 14.6° are observed for the *para* isomer (two crystallographically independent molecules in an asymmetric part of the unit cell), 33.0° in the *meta* derivative and 55.9° for *ortho*-substituted acid. It is worth noting that, for the (2,6-bis(trifluoromethyl)phenyl)boronic acid cocrystals with bifunctional acceptors, this angle is almost 90° [59]. Similar values are observed for the OCF_3_-substituted acids: 16.9° and 23.4° for the *para* isomer and 26.5° for the *ortho* isomer. The last value is lower than for *ortho*-OCF_3_-substituted acid due to the formation of intramolecular hydrogen bond B–O–H···O–CF_3_ [47].

**Figure 4:**
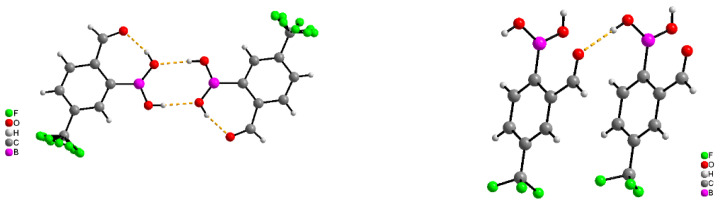
Molecular structures of two isomeric 2-formyl-(trifluoromethyl)phenylboronic acids.

The recently synthesized 4-trifluoromethyl-2-formyl-phenylboronic acid [46] has a similar *syn-anti* conformation. Interestingly, the B(OH)_2_ group is twisted by ca. 100° with respect to the phenyl ring contrary to the isomeric 5-trifluoromethyl-2-formylphenylboronic acid, which is flat [45] (Figure 4). As a consequence, no intramolecular hydrogen bond with the formyl oxygen atom is observed, and each molecule is connected via two types of intermolecular O–H···O hydrogen bonds.

Recently, 5-fluoro-3-morpholin-4-yl-2,1-benzoxaborol-1(3H)-ol and 5-fluoro-3-thiomorpholin-4-yl-2,1-benzoxaborol-1(3H)-ol have been synthesized, and their crystal structures were determined and compared with the parent tavaborole and nonfluorinated benzoxaborole analogs [60]. It was found that the presence of the fluorine atom reduces both the molecular dipole moment, as well as the negative potential at the oxygen atom, in the heterocyclic ring. This effect is increased by the morpholine substituents in the “3” position.

In general, in the crystals of the fluoroboronic compounds, there are no strong interactions involving fluorine atoms. Further supramolecular organization is, however, influenced by weak directional interactions such as C–H···O or C–H···F intermolecular hydrogen bonds, which play a role in the overall crystal packing of the molecules. On the other hand, weakness of the interactions between the large synthons may cause crystals to exhibit a significant disorder or a twinning tendency [43].

## 4. NMR Characterization

In 2014, a paper covering the full NMR spectroscopic characterization (^1^H, ^19^F, ^13^C, ^11^B and ^17^O) of all the fluoro-substituted phenylboronic acids was published [40]. The work includes chemical shifts, as well as coupling constants, and the experimental data are compared with the results of the calculations. Similar data for other fluorinated boronates published up to 2018 were collected in a recent review [15]. The analysis of the presented data shows that ^19^F NMR spectroscopy is an excellent tool for studying structures, equilibria in solutions, complexes or reaction kinetics. The ^19^F chemical shift range exceeds 60 ppm. The change of the chemical shift as a result of structural changes in the molecule is visible even for the peripheral fluorine atoms, which was the basis for the concept of using the “fluorine tag” [61]. The derivatives of the fluorinated boronic acids were used for diol sensing in combination with ^19^F NMR spectroscopy [62,63], as well as in a derivatization protocol for determination of the enantiopurity of sulfonamides [64]. The esterification of bisboronic compounds with fluorinated diols was also studied [65]. Significant differences in the values of coupling constants provide valuable information on the structural parameters. Table 4 shows the NMR spectroscopic data for fluorinated boron compounds published in recent years.

## 5. Equilibria in Solutions

**Scheme 3:**
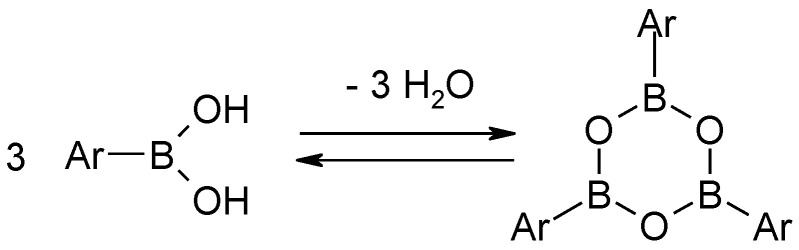
Equilibrium between boronic acid and its anhydride (boroxine).

The formation of cyclic anhydrides by boronic acids, boroxines (Scheme 3), is an important reaction from the point of view of the applications of these compounds.

Among others, this reaction is the basis for the creation of one of the types of covalent organic frameworks (COFs), which are an emerging class of porous covalent organic structures [71]. Dehydration can run by various pathways, depending on the nature and position of the substituents on the arylboronic acid ring [72], and commercial boronic acids are usually a mixture of acid and boroxine. In solutions, the reaction is an equilibrium one, and the shift of this equilibrium depends on the solvent and temperature. For example, the equilibrium constant of this reaction (Scheme 3) in CDCl_3_ is equal to 0.32 M [73], whereas, in THF-d_8_, it is 1.60 × 10^−5^ M [74]. The given values show that, for the solution of acid in THF, the equilibrium is completely shifted to the left, while, for the solution in CDCl_3_, the acid and anhydride are in equilibrium in comparable concentrations. This difference may be due to the different nature of the solvents, of which THF interacts specifically with boronic acid to form hydrogen bonds [75].

The assessment of the anhydride content in a sample of boronic acid and determination of the equilibrium state in the solution is a key issue in the application of these compounds or in physicochemical studies. The suitability of various analytical methods for such evaluations has been tested with the model compound, 3-(trifluoromethyl)phenylboronic acid, which particularly easily dehydrates [75]. It was found that ^19^F NMR spectroscopy is an excellent tool to determine the composition of the fluorine-containing boronic compounds. The conducted research made it possible to explain the discrepancies occurring during previous studies of the solubility of boronic acids in organic solvents [76,77].

**Figure 5:**
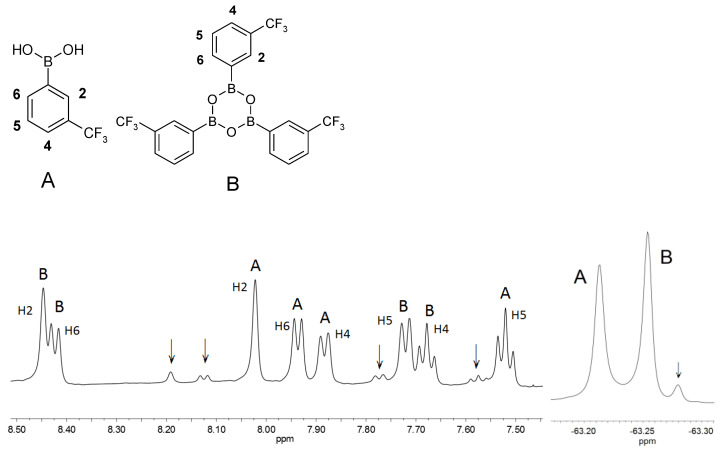
^1^H and ^19^F NMR spectra of a mixture of the acid (**A**) and anhydride (**B**) of *meta*-CF_3_ phenylboronic acid. Low-intensity signals, indicated by arrows, correspond to partially dehydrated species.

The aromatic part of the ^1^H NMR spectra of anhydride and the acid differ significantly. However, the signals are multiplets, and their assignment is not always unambiguous. Moreover, the overlapping of the signals makes their integration difficult. In contrast, ^19^F usually produces narrow signals that are easy to assign and integrate [75]. An example of ^1^H and ^19^F NMR spectra is presented in Figure 5.

**Scheme 4:**
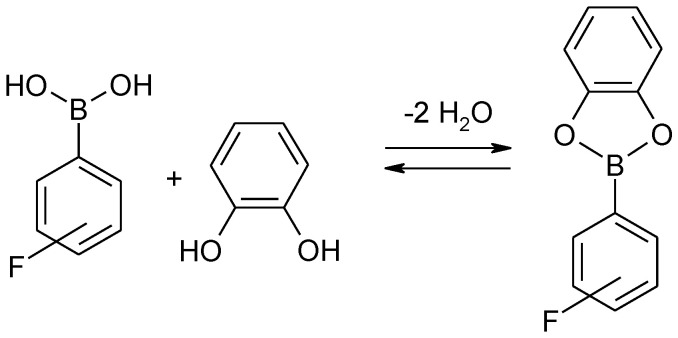
An example of the cyclic ester formation reaction by phenylboronic acid with diol.

Another important equilibrium reaction is the formation of cyclic esters with diols (Scheme 4).

This reaction is the basis of sugars’ binding, as well as catecholamine binding [20], and the equilibrium can be determined by various analytical methods. For fluoro-substituted boronic acids and/or diols, ^19^F NMR spectroscopy again is a quick and accurate analytical tool. As already mentioned in the previous examples, the differences in the ^19^F chemical shifts of the fluorinated acids and esters are so large that the signals are well-separated; moreover, the integration error is lower than in other techniques.

**Scheme 5:**
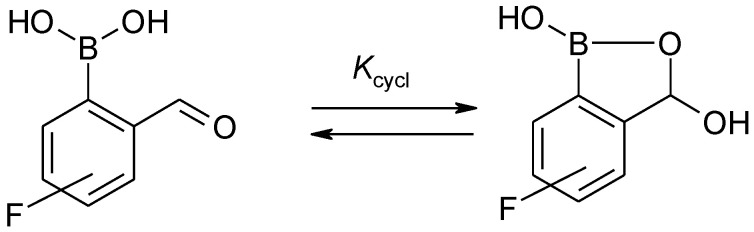
Cyclization of 2-formylphenylboronic acid with the formation of 3-hydroxybenzoxaborole.

^19^F NMR spectroscopy has also been successfully applied to study the equilibrium of the cyclization reaction of fluorinated 2-formylphenylboronic acids (Scheme 5).

Equilibrium constant of this reaction is defined by Equation (2):

(2) $$K_{\mathrm{cycl}}=\frac{\left[B\right]}{\left[A\right]}$$

where [B] stands for the integration of the signal corresponding to the cyclic isomer and [A] for the integration of the formyl group signal. The values of the cyclization constant for fluoro-substituted 2-formylphenylboronic acids determined by Formula 2 in various solvents are presented in Table 5.

**Figure 6:**
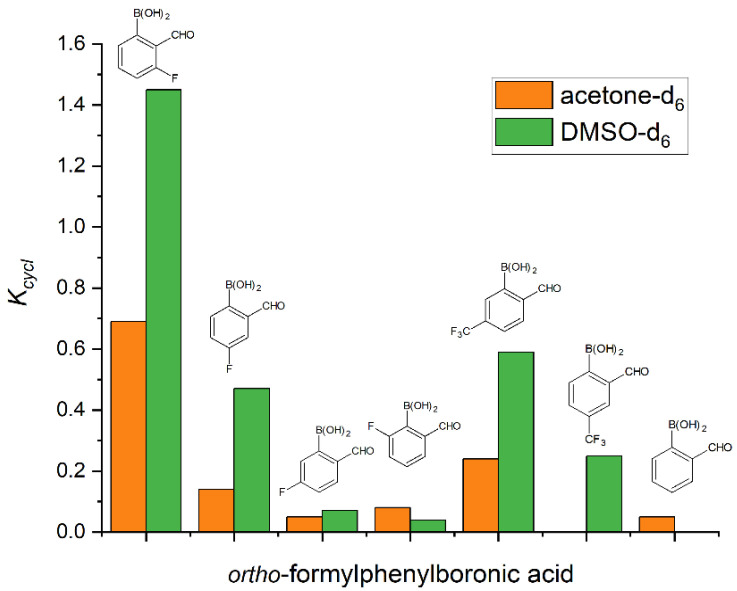
Cyclization constants of selected *ortho*-formylphenylboronic acids in acetone-d_6_ and DMSO-d_6_.

The above data clearly show that the equilibrium of the cyclization reaction strongly depends both on the substituent and its position, as well as on the solvent. The comparison of the values for the fluoro-substituted *ortho*-formylphenylboronic acid isomers in different solvents is shown in Figure 6.

It is worth mentioning that ^17^O NMR spectroscopy was also used to study this reaction [80]. However, this method has significant limitations for practical reasons, e.g., low sensitivity, resulting in a prolonged time for the measurements.

It should be emphasized that ^1^H NMR spectroscopy can be applied only in solutions with solvents that do not contain protons, i.e., mainly in deuterated ones. In contrast, ^19^F NMR spectroscopy allows to study solutions in any solvent, using insert with a sample of standard deuterated solvent.

## 6. Antimicrobial Activity

**Figure 7:**
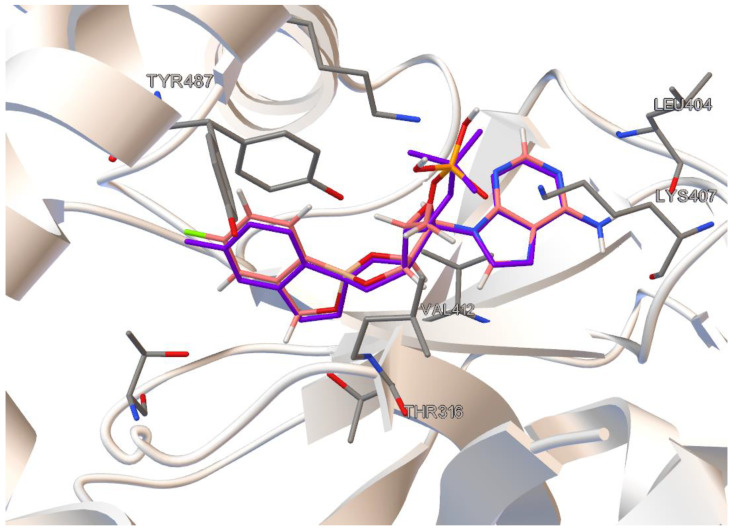
The complex of the AN2690-AMP (Tavaborole) spiroester with the active site of *C. albicans* LeuRS determined in crystal studies (colorful structure) and the optimal structure found in docking procedure (purple structure).

The search for novel antimicrobials is extremely important due to the fact that microorganisms acquire increasing resistance to the commonly used drugs, both antibacterial (antibiotics) as well as antifungal (antimycotics). Fluorinated phenylboronic compounds are amongst the potentially pharmaceutically interesting agents due to the presence of a boronic unit, able to interact with *cis*-diol biomolecules, as well as a wide abundance of fluorine substituents among the currently used drugs. It is worth noting that the first phenylboronic antifungal drug approved by FDA in 2014 also contains the fluorine atom (Tavaborole, AN2690, Kerydin). The generally accepted mechanism of action of the Tavaborole drug is based on crystallographic studies and relies on the formation of a spiroester with adenosine monophosphate (AMP) in the editing site of LeuRS in *Thermus thermophilus* [13] and *Candida albicans* [81] fungi. As part of the struggle for novel antifungal drugs, a model system was developed, enabling a comparison of interactions of various phenylboronic compounds and the lead drug (Tavaborole) with that enzyme in silico [45]. Interestingly, although Tavaborole contains a fluorine atom, no specific interactions of that substituent with the enzyme were detected either in the crystal structure [81] of the complex or in the docking studies (Figure 7) [45].

In the docking studies of the interactions of phenylboronic compounds with selected enzymes (*C. albicans* LeuRS and *E. coli* LeuRS), it was determined that some of the compounds bind tightly with the enzymes, which may result in their antifungal activity [45,47,82]. It was also shown that the optical isomerism of 3-substituted benzoxaboroles displays a significant impact on the determined binding energies and inhibition constants (*R*-enantiomers dominate amongst the strongest binding ligands) [82]. However, it should be emphasized that the arrangement of the tested boronic ligands in the active center of the enzyme is, in most cases, different from that known for Tavaborole’s spiroester determined in structural studies [13,45]. This, in turn, may result in a reduced activity of the studied derivatives in comparison with the lead drug determined in in vitro studies of the activity against *C. albicans* [47,83]. Docking studies showed also that the hydrogen bond interactions with the active sites of the enzyme takes place in the AMP part of the spiroester and not in the benzoxaborole part (Figure 7).

**Figure 8:**
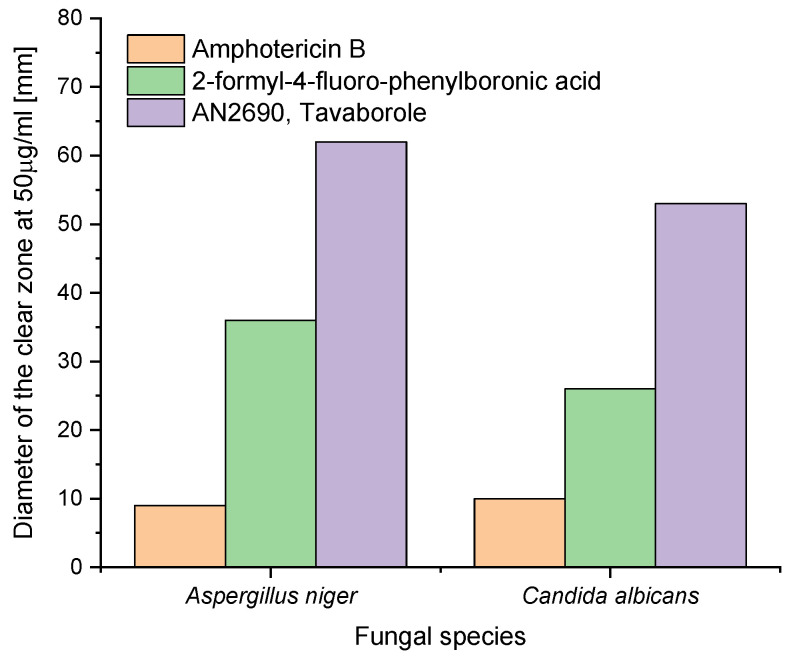
Diameters of the zone of totally inhibited the growth of fungal strains (mm) for amphotericin B (orange), 2-formyl-fluoro-phenylboronic acid (green) and Tavaborole (violet) applied at a 50-µg/mL concentration.

The antimicrobial activity of over a dozen fluorine-containing phenylboronic compounds against several species of fungi, as well as Gram-positive and Gram-negative model bacteria, were recently examined in vitro. The evaluated antimicrobial action was compared with the activity of the reference compounds, including standard antibiotics and/or Tavaborole. The initial assessment was performed on the basis of the well tests, showing that some of the investigated fluorinated boronic derivatives displayed much higher diameters of the zone of inhibited growth of *Aspergillus niger* and *Candida albicans* species in comparison with amphotericin B (Figure 8). The results confirmed not only reasonable activity but also high diffusion of the studied compounds within the applied solid medium.

For substances exerting antimicrobial activity in preliminary well tests, the Minimal Inhibitory Concentration (MIC) values were determined [38,45,47,68,70,79,83]. Several structural elements that determine the antimicrobial activity of the tested compounds were indicated. It was shown that the presence of a heterocyclic benzoxaborole system is essential for the antifungal activity. Contrary, analogous phenylboronic acids do not show such an action [68,84,85]. The only exception are 2-formyl phenylboronic compounds, which actually exert antifungal activity, which was studied against several fungal species, such as: *A. niger, A. terreus, F. solani, P. ochrochloron, C. albicans* and *C. tenuis* (Table 6) [79].

The explanation of this feature for 2-formylphenylboronic acids was associated with the presence of their cyclic tautomers existing in a solution in equilibrium with the keto-form (Scheme 5) [79].

A similar effect was observed for CF_3_-substituted 2-formylphenylboronic acids [45,46]. In addition to antifungal activity, the investigated compounds showed reasonable antibacterial activity against *B. cereus* [45,46], while other phenylboronic acids, as well as most of the studied benzoxaboroles, do not show such an activity.

Tests of antifungal properties of fluoro-substituted benzoxaboroles show that the introduction of an amino substituent in the “3” position of the heterocyclic ring is associated with a reduced activity of those compounds towards *C. albicans*, compared to benzoxaboroles without these substituents [83].

The presence and position of the fluorine substituent also have a significant influence on the activity against *C. albicans* [83]. Compounds containing a fluorine atom in the benzoxaborole ring at a position analogous to that in Tavaborole usually display reasonably higher antifungal activity in comparison to those without fluorine substituents or with such a substituent at a different position [38,83]. The influence of Tavaborole, as well as other benzoxaboroles, on the growth and composition of photosynthetic pigments in cyanobacteria (*Chroococcidiopsis thermalis, Arthrospira platensis, A. fusiformis, A. maxima* and *Anabaena*) was also investigated. It was shown that the investigated compounds changed the quantitative relationships between the contents of chlorophylls, phycobiliproteins and carotenoids within those microorganisms [86]. A mechanochemical method of fluorinated bis(benzoxaboroles) synthesis was recently developed [70]. It was found that the antifungal activity of the obtained bis(benzoxaboroles) depends on the position of the fluoro substituent in relation to the boron atom [70]. As in the case of other investigated derivatives, the presence of a fluorine substituent in the para position in relation to the boron atom results in increased antifungal activity [38,70,79,83].

Recently, the antibacterial activity of three isomers of trifluoromethoxyphenylboronic acids was investigated [47]. Despite the promising results of docking studies, none of the studied compounds revealed considerable activity against *Escherichia coli*. At the same time, the *meta* and *para* isomers showed limited activity against *Bacillus cereus*, while the *ortho* isomer was inactive.

Recently, it was found that 2-fluoro-6-formylphenylboronic acid and 3-morpholino-5-fluorobenzoxaborole exhibit strong cell cycle arrest induction in G2/M associated with caspase-3 activation in an A2780 ovarian cancer cell line [87].

Several examples of antiviral drugs containing both fluorine and boronic substituents have been reported in a recent review [88]. However, only a few of these compounds contain both substituents on the same fragment, which is crucial for their interaction.

## 7. Conclusions

The introduction of fluorine substituents into boronic structures influences their properties considerably. Fluorine substituents increase the acidity of boronic acids. This effect is dependent on the position of the substituent: the strongest one is observed for the *ortho* position, which is due to the formation of an intramolecular B–O···F hydrogen bond. For the *para* position, the rise in acidity is smaller due to the compensation of the inductive and mesomeric effects. Similar regularities exist for benzoxaboroles. The presence of further fluoro substituents increases the acidity, but the effect is dependent on the position of the substituents. The CF_3_ and OCF_3_ substituents at the *meta* and *para* positions increase their acidity to a greater extent than the F-substituent. On the contrary, the presence of both groups at the *ortho* positions results in a drop of Lewis acidity due to the steric effect, hindering the formation of the boronate anion.

The influence of the fluorine substituents on the acceptor number of boronate esters is similar to that on the acidity constant of the corresponding acids. However, the correlation of these values is moderate due to the differences in the steric effects of the hydroxyl anion in the case of p*K*_a_ and triethylphosphine oxide for the acceptor number.

The presence of fluorinated substituents significantly influences molecular and crystal structures. The majority of fluoro-substituted boronic acids constitute the most common dimeric synthons with the *syn-anti* arrangement of hydroxyl groups. Differentiation of the crystal structure occurs at the higher-order supramolecular level, in which weak intermolecular C–H···F and C–H···O interactions play the main role. A single fluorine atom at the *ortho* position forms a weak intramolecular B–O···F bond, but the second *ortho*-F substituent no longer forms such a bond. Both the CF_3_ and OCF_3_ groups at the *ortho* position cause a significant rise in the dihedral angle between the boron group and the phenyl ring plane. For the two *ortho*-CF_3_ groups in one molecule, this angle is equal to 90°.

^19^F NMR spectroscopy is an excellent tool for studying structures, equilibria in solutions, complexes or reaction kinetics of fluorinated phenylboronic acids. This is due to the wide range of chemical shifts (ca. 60 ppm), resulting in good signal separation and relatively easy analysis of the coupling constants.

Boronic acids in solutions are in equilibrium with the corresponding boroxins. This equilibrium is dependent on the substituents, as well as on the solvent. *Ortho*-formylphenylboronic acids show a tautomeric equilibrium with the formation of the corresponding 3-hydroxybenzoxaboroles, which was investigated by ^19^F NMR.

In the docking studies of the interactions of fluoro-substituted phenylboronic compounds, it was found that some phenylboronic compounds bind tightly with the enzymes, which may result in their antifungal activity. Interestingly, no specific interactions of the fluorine substituents with the enzyme were detected.

The antimicrobial activity of fluorine-containing phenylboronic compounds against several species of fungi, as well as Gram-positive and Gram-negative model bacteria, was compared with the activity of the reference compounds. It was found that some of the investigated fluorinated boronic derivatives display a much higher inhibition growth of *Aspergillus niger* and *Candida albicans* species in comparison with amphotericin B. The equilibrium of the tautomerization of 2-formylphenylboronic compounds leading to 3-hydroxybenzoxaboroles plays the key role in this activity.

## Figures and Tables

**Table 1:** Hammett constants *σ* for fluoro-substituents.

Substituent	*σ_meta_*	*σ_para_*
F	0.34	0.06
CF_3_	0.43	0.54
OCF_3_	0.38	0.35

**Table 2:** p*K*_a_ values of boronic acids X-C_6_H_4_B(OH)_2_ with F, CF_3_ and OCF_3_ substituents.

X	*ortho*	*meta*	*para*	Reference
F	7.89 ^a^	8.09	8.77	[36]
	*7.85* ^b^	*8.15*	*8.71*	
CF_3_	9.45	7.88	7.82	[43]
	*9.58*	*7.85*	*7.90*	
	-	7.87	7.39	[44]
2-CHO, 5-CF_3_	5.67			[45]
2-CHO, 4-CF_3_	5.75			[46]
OCF_3_	9.51	7.79	8.11	[47]
	*9.49*	*7.96*	*8.03*	

^a^ Values obtained by the spectrophotometric method. ^b^ Values obtained by the potentiometric method (in italics).

**Table 3:** p*K*_a_ values of benzoxaboroles with F and CF_3_ substituents.

Compound		Position				Reference
		4	5	6	7	
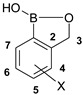	X = F	6.36	6.97	6.57	7.42	[38]
		6.45	-	6.63	-	[39]
	X = CF_3_	-	6.28	-	-	[46]
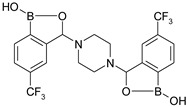		5.54				[46]

**Table 4:** ^19^F NMR chemical shifts of recently described fluoro-substituted boronic compounds.

Compound	Chemical Shift/ppm	Solvent	Reference
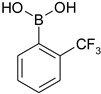	−59.37	CDCl_3_	[43]
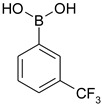	−62.92	CDCl_3_	[43]
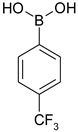	−63.39	CDCl_3_	[43]
	−61.1	CD_3_OD	[66]
	−61.8	DMSO−d_6_	[67]
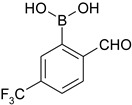	−63.44	CDCl_3_	[45]
	−63.29	C_6_D_6_	[45]
	−62.94 to −61.98 ^a^	acetone-d_6_	[45]
	−61.67 to −60.73 ^a^	DMSO−d_6_	[45]
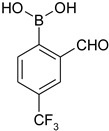	−68.11	CDCl_3_	[46]
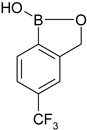	−63	acetone-d_6_	[46]
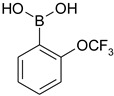	−56.93	acetone-d_6_	[47]
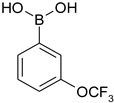	−57.64	acetone-d_6_	[47]
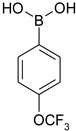	−57.59	acetone-d_6_	[47]
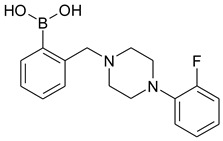	−124.1	acetone-d_6_/D_2_O	[68]
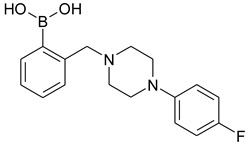	−124.92	acetone-d_6_	[68]
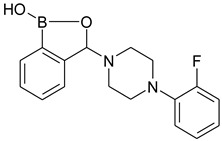	−122.49	acetone-d_6_	[68]
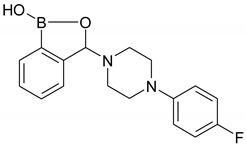	−121.37	acetone-d_6_	[68]
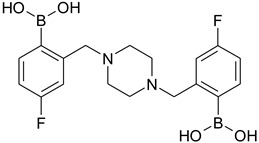	−116.56	CD_3_OD	[69]
^ 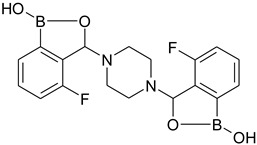 ^	−118.96	DMSO−d_6_	[70]
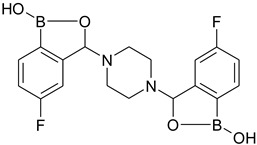	−109.44 ^b^	CD_3_OD	[69]
	−109.55 ^b^		
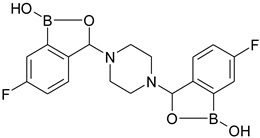	−115.02	DMSO−d_6_	[70]
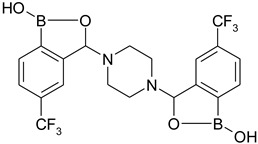	−60.97 ^b^	acetone-d_6_	[46]
	−61.02 ^b^		

^a^ Cyclic tautomeric form. ^b^ Diastereomers.

**Table 5:** Cyclization constants of fluorinated 2-formylphenylboronic acids.

Compound	Solvent	*K*_cycl_	Method	Temperature/K	Ref.
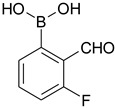	acetone-d_6_	0.94	^1^H NMR	303	[78]
	acetone-d_6_	0.69	^1^H NMR	298	[27]
	DMSO-d_6_	1.45	^1^H NMR	298	[78]
	DMSO-d_6_	1.46	^19^F NMR	298	[79]
	DMSO-d_6_/D_2_O	1.35	^19^F NMR	298	[79]
	D_2_O	0.17	^1^H NMR	303	[78]
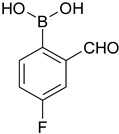	acetone-d_6_	0.14	^1^H NMR	303	[78]
	DMSO-d_6_	0.47	^19^F NMR	298	[79]
	DMSO-d_6_/D_2_O	0.49	^19^F NMR	298	[79]
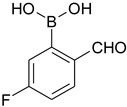	acetone-d_6_	0.05	^1^H NMR	298	[27]
	DMSO-d_6_	0.07	^19^F NMR	298	[79]
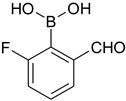	acetone-d_6_	0.08	^1^H NMR	298	[27]
	DMSO-d_6_	0.04	^19^F NMR	298	[79]
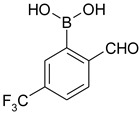	DMSO-d_6_	0.59	^1^H, ^19^F NMR	298	[45]
	acetone-d_6_	0.24	^1^H, ^19^F NMR	298	[45]
	D_2_O	0.26	^1^H, ^19^F NMR	298	[45]
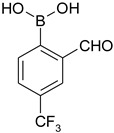	DMSO-d_6_	0.25	^19^F NMR	298	[46]

**Table 6:** MIC values of fluorine-substituted 2-formylphenylboronic acids (µg/mL).

Fungus	Substituent/MIC (µg/mL)			
	3-F	4-F	5-F	6-F
*A. niger*	31.3	2	62.5	<125
*A. terreus*	31.3	<1	31.3	<125
*F. solani*	15.6	15.6	31.3	<125
*P. ochrochloron*	31.3	7.8	31.3	125
*C. albicans*	62.5	62.5	62.5	125
*C. tenuis*	62.5	125	62.5	62.5

## Data Availability

Not applicable.
